# Normal distribution of H3K9me3 occupancy co-mediated by histone methyltransferase BcDIM5 and histone deacetylase BcHda1 maintains stable ABA synthesis in *Botrytis cinerea* TB-31

**DOI:** 10.3389/fmicb.2024.1339576

**Published:** 2024-03-04

**Authors:** Zhao Wei, Dan Shu, Xiaonan Hou, Tianfu Li, Zhemin Li, Di Luo, Jie Yang, Hong Tan

**Affiliations:** ^1^CAS Key Laboratory of Environmental and Applied Microbiology, Environmental Microbiology Key Laboratory of Sichuan Province, Chengdu Institute of Biology, Chinese Academy of Sciences, Chengdu, China; ^2^University of the Chinese Academy of Sciences, Beijing, China

**Keywords:** *Botrytis cinerea*, H3K9me3, abscisic acid, secondary metabolism, gene regulation

## Abstract

Abscisic acid (ABA) is a conserved and important “sesquiterpene signaling molecule” widely distributed in different organisms with unique biological functions. ABA coordinates reciprocity and competition between microorganisms and their hosts. In addition, ABA also regulates immune and stress responses in plants and animals. Therefore, ABA has a wide range of applications in agriculture, medicine and related fields. The plant pathogenic ascomycete *B. cinerea* has been extensively studied as a model strain for ABA production. Nevertheless, there is a relative dearth of research regarding the regulatory mechanism governing ABA biosynthesis in *B. cinerea*. Here, we discovered that H3K9 methyltransferase BcDIM5 is physically associated with the H3K14 deacetylase BcHda1. Deletion of *Bcdim5* and *Bchda1* in the high ABA-producing *B. cinerea* TB-31 led to severe impairment of ABA synthesis. The combined analysis of RNA-seq and ChIP-seq has revealed that the absence of BcDIM5 and BcHda1 has resulted in significant global deficiencies in the normal distribution and level of H3K9me3 modification. In addition, we found that the cause of the decreased ABA production in the Δ*Bcdim5* and Δ*Bchda1* mutants was due to cluster gene repression caused by the emergence of hyper-H3K9me3 in the ABA gene cluster. We concluded that the ABA gene cluster is co-regulated by BcDIM5 and BcHda1, which are essential for the normal distribution of the *B. cinerea* TB-31 ABA gene cluster H3K9me3. This work expands our understanding of the complex regulatory network of ABA biosynthesis and provides a theoretical basis for genetic improvement of high-yielding ABA strains.

## Introduction

Abscisic acid (ABA) is a sesquiterpene signaling molecule produced in all kingdoms of life. Different organisms have developed different biosynthesis and signal transduction pathways related to ABA (Lievens et al., 2017). To date, the best-known functions of ABA are derived from its role as a major plant hormone in a variety of plant processes, including plant growth, seed dormancy and fruit ripening among others (Singh and Roychoudhury, 2023). Exogenous application has important effects on the promotion of plant growth and development, improvement of stress tolerance and increase in disease resistance (Zehra et al., 2021). In addition, ABA plays an important role in compatible mutualistic interactions such as mycorrhizal and rhizosphere bacteria in plants (Stec et al., 2016). In humans and animal models, studies have shown that the nutrient-derived ABA or ABA treatment is beneficial in the treatment of inflammatory diseases such as colitis and type 2 diabetes, which confer potential to ABA as an interesting nutraceutical or pharmacognostic drug. Meanwhile, the anti-inflammatory, anti-apoptotic, and anti-oxidative properties of ABA in humans and animal models have sparked an interest in this molecule and its signaling pathway as a novel pharmacological target (Sakthivel et al., 2016; Lievens et al., 2017; Shabani et al., 2023). All these points indicate that ABA has a broad application prospect in agriculture and medicine. At present, ABA has been industrialized through *B. cinerea* fermentation. *B. cinerea* is a typical phytopathogenic ascomycete that causes gray mold in over 1,000 host plants annually, but it is also a reservoir of secondary metabolites (Dean et al., 2012). Currently, the *B. cinerea* T4 and B05.10 genome sequences predicted 43 genes probably encoding key enzymes (KEs) for secondary metabolite (SM) biosynthesis (Amselem et al., 2011), including 21 polyketide synthases (PKS), 1 chalcone synthase, 6 sesquiterpene cyclases (STC), 9 non-ribosomal peptide synthases (NRPS), 2 dimethylallyl tryptophan synthases (DMATS), and 5 diterpene cyclases (DTC), suggesting that in addition to ABA, *B. cinerea* has the potential to produce the above SMs.

In our laboratory, the *B. cinerea* wild-type strain TBC-6 was isolated from wheat stems and leaves in southwest China. *B. cinerea* TBC-6 improvements were performed and generated a series of *B. cinerea* mutants with different yields of ABA (Gong et al., 2014), such as TB-31, TB-3-H8, and TBC-A, which were used for biosynthesis regulation research and industrial production of ABA (Ding et al., 2016). The ABA biosynthesis gene cluster and positive regulator in *B. cinerea* have been identified, including two hypothetical P450 monooxygenase coding genes *Bcaba1* and *Bcaba2*, hypothetical FPP (Farnesyl Pyrophosphate) catalysis gene *Bcaba3*, presumptive short-chain dehydrogenase/reductase coding genes *Bcaba4*, and pathway-specific transcription factors *BcabaR1* (Wang et al., 2018). Meanwhile, we found that the global regulator BcLAE1 is involved in the regulation of ABA biosynthesis. Interestingly, H3K9me3 was significantly altered in the BcLAE1 knockout mutant. We therefore suspect that ABA synthesis is regulated by higher-level epigenetic regulation in addition to pathway-specific transcription factors and global regulators.

Epigenetic regulation refers to heritable regulation independent of DNA sequence changes caused by RNA interference, DNA methylation, and Posttranslational modification of histones (Lai et al., 2022). Among them, histone posttranslational modification affects chromatin modification status (euchromatin and heterochromatin) through phosphorylation, methylation and acetylation of histone N-terminal tail, thus realizing the regulation of gene expression (Bind et al., 2022). Studies have shown that monomethylation, dimethylation, and trimethylation (me1/2/3) of histone lysine are catalyzed by histone lysine methyltransferase (KMT), mostly with a catalytic Set (Su(var)3–9, enhancer-of-zeste and trithorax) domain, which transfer a methyl group from S-Adenosyl-L-Methionine (SAM) to the lysine residues on the N-terminal of H3 or H4 (Brosch et al., 2008). Changes in chromatin structure achieved by histone methylation play a key role in regulation of fungal SM biosynthesis. For example, H3K4me2/3, H3K36me1/3 and H4K20me1 are commonly associated with transcriptional activation, whereas H3K9me2/3, H3K27me2/3 and H4K20me3 are associated with transcriptional repression (Lai et al., 2022). The H3K4me2/3 alteration in *Fusarium Fujikuroi* encouraged the expression of the Gibberellin (GA) gene cluster and increased GA production (Janevska et al., 2018). Deletion of FgKMT6 in *Fusarium graminearum* results in a genome-wide deficiency of H3K27me3, which activates the expression of mycotoxins, pigments, and some secondary metabolism-related genes in the Δ*FgKMT6* (Tang et al., 2021). The methylation modification of H3K36 in *Aspergillus flavus* is caused by the AshA and SetB, which can directly control the synthesis of mycotoxins (Zhuang et al., 2022). However, the mechanism by which histone modifications regulate ABA biosynthesis in *B. cinerea* has not been reported.

In the current study, we discovered the histone H3K9 methyltransferase BcDIM5 is involved in the regulation of ABA synthesis. This regulatory role is achieved by synergizing with the H3K14 deacetylase BcHda1. Loss of BcDIM5 and BcHda1 led to an excessive accumulation of H3K9me3 occupancy in the ABA gene cluster, resulting in a severe impairment of ABA production. Furthermore, our observations implied that BcHda1 and BcDIM5 are required for normal distribution of constitutive heterochromatic modifications and the stable maintenance of ABA biosynthesis in *B. cinerea* TB-31.

## Results

### BcDIM5 is required for ABA production in *Botrytis cinerea* TB-31

**Figure 1:**
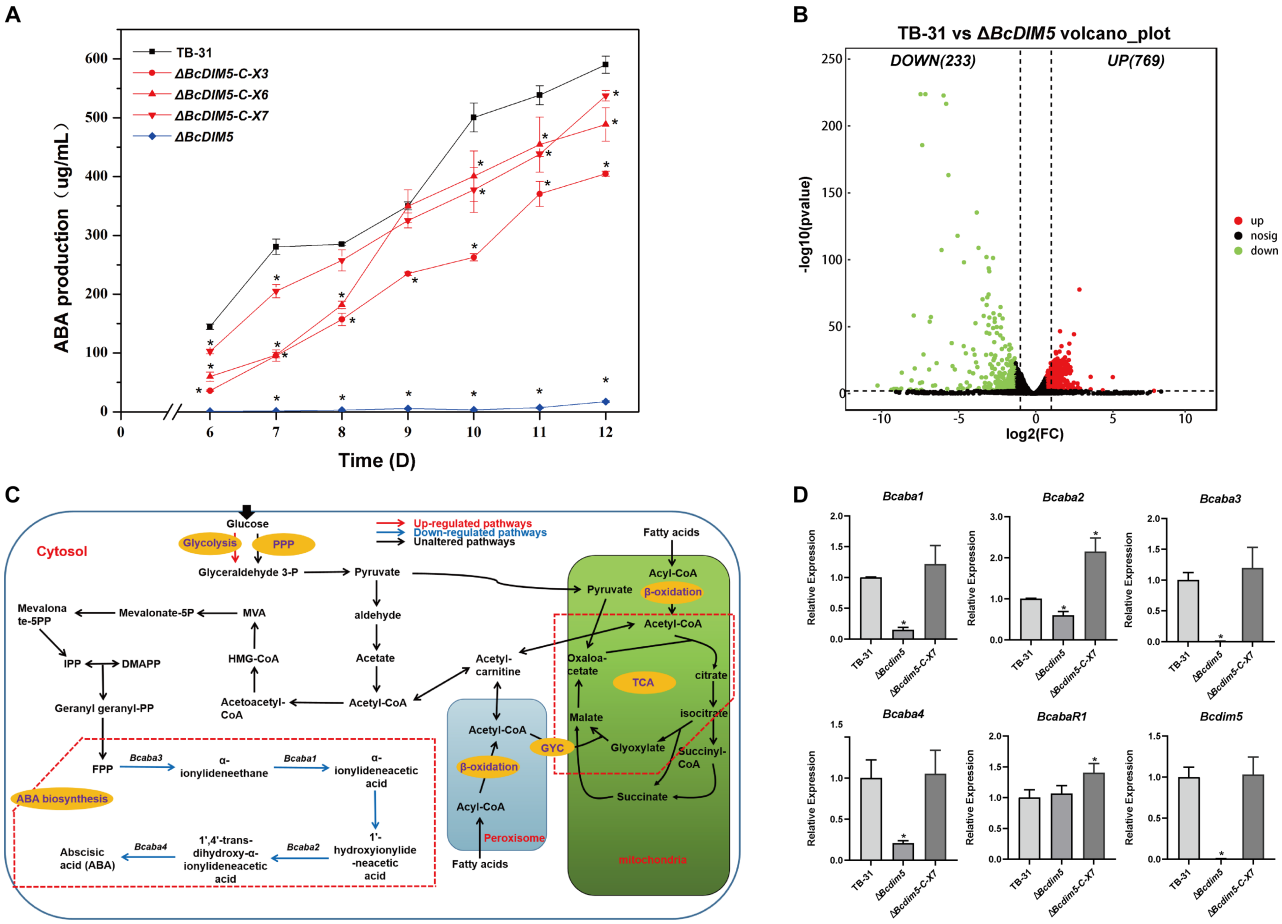
*Bcdim5* is required for ABA production in *B. cinerea* TB-31. **(A)** ABA production of control strain TB-31, Δ*Bcdim5* and three randomly selected Δ*Bcdim5*-C mutants at different culture time. Samples for the quantitative determination of ABA production were collected at 6–12 days. The error bars indicate the standard errors of the mean for three replicate cultures (*n* = 3). Asterisks indicate significant differences in ABA production between selected mutants and TB-31 (*p* < 0.05). **(B)** DEGs between TB-31 and Δ*Bcdim5* on day 6 of PDA culture. The criteria for the selection of DEGs were a log2 |fold change| ≥ 1, value of *p* < 0.05, and RPKM of at least one sample larger than 1. **(C)** Schematic of metabolic pathways associated with ABA production affected by *Bcdim5* deletion. The red arrows indicate upregulated pathways. Blue arrows indicate downregulated pathways, and black arrows indicate pathways that were not observably regulated (log2 |fold change| ≥ 1, value of *p* < 0.05 and RPKM of at least one sample larger than 1). The red dashed box annotates the glyoxylic acid cycle (GYC) pathway. **(D)** RT-qPCR examining the transcriptional levels of ABA gene cluster, *BcabaR1* and *Bcdim5* in Δ*Bcdim5*, Δ*Bcdim5*-C-X7, and TB-31. The relative transcriptional levels of selected genes were obtained after normalization to the constitutive tubulin reference gene (BC1G_05600) at 6 days. The relative values for selected genes transcription at 6 days in TB-31 were assigned as 100%. Shown are means and SEM, *n* = 3 independent biological replicates. *p < 0.05 versus the same genes of the TB-31 group.

In a previous study, we discovered that the global regulator BcLae1 knockdown significantly reduced the production of ABA and changed the global H3K9me3 modification (Wei et al., 2022). Therefore, we speculated whether changes in H3K9me3 modification would have an effect on ABA synthesis. Based on this, we knocked out the reported H3K9 methyltransferase BcDIM5 in *B. cinerea* TB-31 (Supplementary Figure S1). In comparison to TB-31, the ABA production of Δ*Bcdim5* was reduced by more than 97% during the 6–12 days culture period. ABA production was recovered following *Bcdim5* complementation. At day 12, the randomly selected complement strains Δ*Bcdim5*-C-X3, X6 and X7 restored ABA yield to more than 69% of that of TB-31 (Figure 1A), demonstrating that BcDIM5 is involved in the regulation of ABA synthesis.

When *Bcdim5* is knocked out, ABA synthesis is severely reduced from early to late stages. To understand the impact of *Bcdim5* knockout on the related pathway of ABA synthesis, we selected the early stage of ABA synthesis for RNA-seq analysis. Based on the results of differential analysis, the differentially expressed genes (DEGs) were identified between the transformants using the threshold log2 |fold change| ≥ 1, *p*-value <0.05 and RPKM value of at least one sample larger than 1. And a total of 1,002 DEGs were obtained, of which 769 genes were up-regulated and 233 genes were down-regulated (Figure 1B).

Subsequently, we focused on analyzing changes in the expression of ABA synthesis-related pathways, including carbohydrate transport and utilization, acetyl-CoA synthesis and transport-related pathways, FPP synthesis and ABA synthesis gene cluster (Supplementary Dataset S1). It was found that the transcription levels of 1,4-Beta-mannopyranoside mannosidase *manA* (BCIN_02g07520), Arabinoglucosidase *xylB* (BCIN_08g00060) and Ferulic acid esterase *faeb*-2 (BCIN_09g05740) were reduced. The expression of the remaining enzymes involved in sugar transporters, degrading enzymes and permeases were not significantly altered. This result suggests that the carbon source utilization of Δ*Bcdim5* was not significantly changed compared with TB-31. The central metabolite acetyl-CoA is the link between the primary metabolic pathway and the secondary metabolic pathway, which is also the precursor of the synthesis of ABA in *B. cinerea*. Pyruvate is an important node in glucose synthesis of acetyl-coA. Both glycolysis and pentose phosphate pathway (PPP) convert glucose into intracellular pyruvate. However, there was no discernible variation in the transcription levels of the genes involved in the PPP. The expression levels of BCIN_15g04970 (glucose-6-phosphate isomerase) and BCIN_08g06300 (6-phosphofructose kinase subunit α) genes in glycolysis pathway were higher than that of TB-31, which ensured that the glucose catabolism of Δ*Bcdim5* was not inhibited. The expression levels of associated genes in the β-oxidation pathway, which generates acetyl-coA from fatty acids, did not differ significantly either. The transport of acetyl-coA involves the TCA cycle and carnitine shuttle system, but the alterations in gene expression pertaining to these transport systems were not significant. Consequently, the decrease in ABA production in Δ*Bcdim5* cannot be attributed to the synthesis and transport of acetyl-coA. Additionally, the transcription levels of enzymes involved in the MVA pathway remain unchanged, while the transcription level of the ABA synthesis gene cluster (*Bcaba1, Bcaba2*, *Bcaba3* and *Bcaba4*) experienced a significant reduction in the Δ*Bcdim5* (Figure 1C). Therefore, we suggested that this is the direct cause of the reduced ABA production in Δ*Bcdim5*, which was confirmed by RT-qPCR (Figure 1D).

### Histone deacetylase BcHda1 interacts with BcDIM5 and participates in the regulation of ABA synthesis

**Figure 2:**
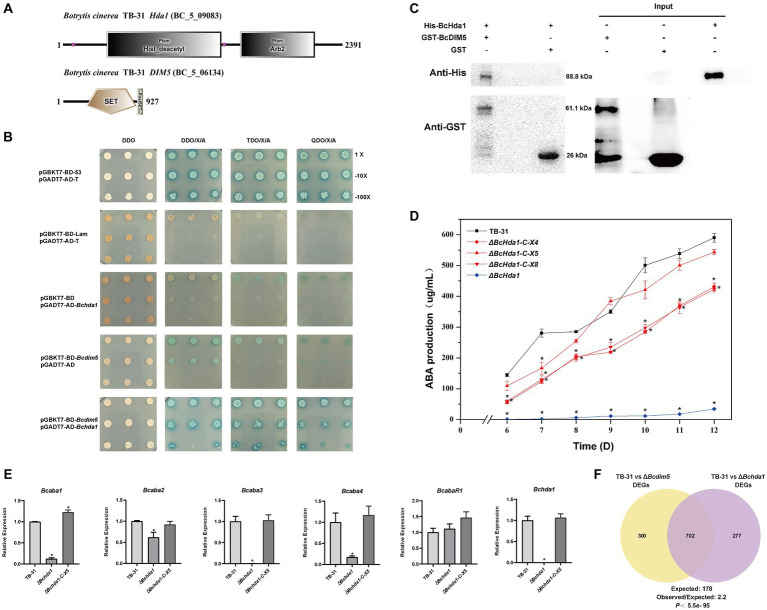
The interacting protein BcHda1 of BcDIM5 is also involved in ABA synthesis. **(A)** Structures and conserved domains of BcHda1 and BcDIM5. The indicated domains include: SET (IPR001214), Post-SET (IPR003616), His_deacetylse (IPR023801), Arb2 (IPR019154). **(B)** Yeast-two-hybrid assay between BcHda1 and BcDIM5. pGBKT7-BD-53/pGADT7-AD-T was the positive control, pGBKT7-BD-lam/pGADT7-AD-T, pGBKT7-BD/pGADT7-AD-*Bchda1*, and pGBKT7-BD- *Bcdim5*/pGADT7-AD were the negative controls. The experimental group pGBKT7-BD-*Bcdim5*/pGADT7-AD-*Bchda1* could grow in TDO/X/A and QDO/X/A medium after hybridization. **(C)** GST-Pulldown validates the interaction of BcDIM5 with BcHda1. **(D)** ABA production of control strain TB-31, Δ*Bchda1* and three randomly selected Δ*Bchda1*-C mutants at different culture time. Samples for the quantitative determination of ABA production were collected at 6–12 days. The error bars indicate the standard errors of the mean for three replicate cultures (*n* = 3). Asterisks indicate significant differences in ABA production between selected mutants and TB-31 (*p* < 0.05). **(E)** RT-qPCR examining the transcriptional levels of ABA gene cluster, *BcabaR1* and *Bchda1* in Δ*Bchda1*, Δ*Bchda1*-C-X5, and TB-31. The relative values for selected genes transcription at 6 days in TB-31 were assigned as 100%. Shown are means and SEM, *n* = 3 independent biological replicates. *p < 0.05 versus the same genes of the TB-31 group. **(F)** Venn diagram showing the significant overlap of DEGs in the Δ*Bcdim5* and Δ*Bchda1*. *p*-values for the significance of the overlap between gene sets were calculated using Fisher’s exact test.

In eukaryotes, histone modifying enzymes commonly bind to multiple subunits to affect chromatin homeostasis (Beisel and Paro, 2011; Song et al., 2015). The DIM-5 complex has been well characterized in fission yeast. In addition to forming a methyltransferase complex with RiK1, Raf1, Raf2, and Pcu4 subunits (Oya et al., 2019; Shan et al., 2021), DIM-5 exists in close association with the Argonaute complex to maintain heterochromatin assembly (Buker et al., 2007). In the *B. cinerea* genome, the Arb2 subunit of the Argonaute complex was found to be present in the putative H3K14 deacetylase BcHda1 by NCBI B_LAST_ comparison (Figure 2A). We therefore speculated BcHda1 as a potential interacting partner of BcDIM5. Based on this, Y2H assays were performed on BcDIM5 and BcHda1, revealing that the yeast cells were capable of growing on the TDO/X/A plate and QDO/X/A plate following the interaction of BcHda1 and BcDIM5 (Figure 2B), suggesting a potential functional relationship between the two proteins. Additionally, the physical association of BcHda1 and BcDIM5 was confirmed *in vitro* through GST-pulldown assay after the purification of His-BcHda1 and GST-BcDIM5 (Figure 2C), suggesting a potential functional relationship between the two proteins.

Subsequently, we also performed knockdown experiments on BcHda1. Knockdown of *Bchda1* revealed that the ABA production of Δ*Bchda1* was similar to that of Δ*Bcdim5*. During the 6–12 days culture period, ABA produced by Δ*Bchda1* was reduced by more than 94% compared to TB-31. The ABA yield was restored upon *Bchda1* complementation, particularly in Δ*Bchda1*-C-X5, which approached that of the control strain TB-31 (Figure 2D). These findings suggested that *Bchda1* is also essential for the ABA biosynthesis in *B. cinerea* TB-31. The effect of *Bchda1* knockdown on the ABA synthesis pathway was assessed by RNA-seq analysis (Supplementary Table S2). The results showed that except for a significant decrease in the transcription levels of the ABA synthesis gene cluster (Figure 2E), the expression changes of the other related pathways were not significant, implying that BcDIM5 and BcHda1 affect ABA synthesis by regulating the transcription levels of the ABA gene cluster. Comparison of RNA-seq data for Δ*Bchda1* and Δ*Bcdim5* revealed a large number of identical terms in the GO and KEGG enrichment results (Supplementary Figure S2). In particular, the expression of many DNA repair genes was upregulated, suggesting that knockdown of *Bcdim5* and *Bchda1* may have a significant impact on growth and development. From the phenotypic results we clearly observed that the growth of Δ*Bchda1* and Δ*Bcdim5* became retarded compared to the control strain TB-31 (Supplementary Figure S3). Meanwhile, the two sets of transcriptome data exhibit 702 identical DEGs, which constitute 71.7 and 70.1% of the total DEGs of TB-31 vs. Δ*Bchda1* and TB-31 vs. Δ*Bcdim5*, respectively (Figure 2F). The overlap of the DEGs data demonstrates that the two proteins act synergistically to exert regulatory functions.

### Overexpression of *Bcdim5* and *Bchda1* did not increase ABA production in *Botrytis cinerea* TB-31

**Figure 3:**
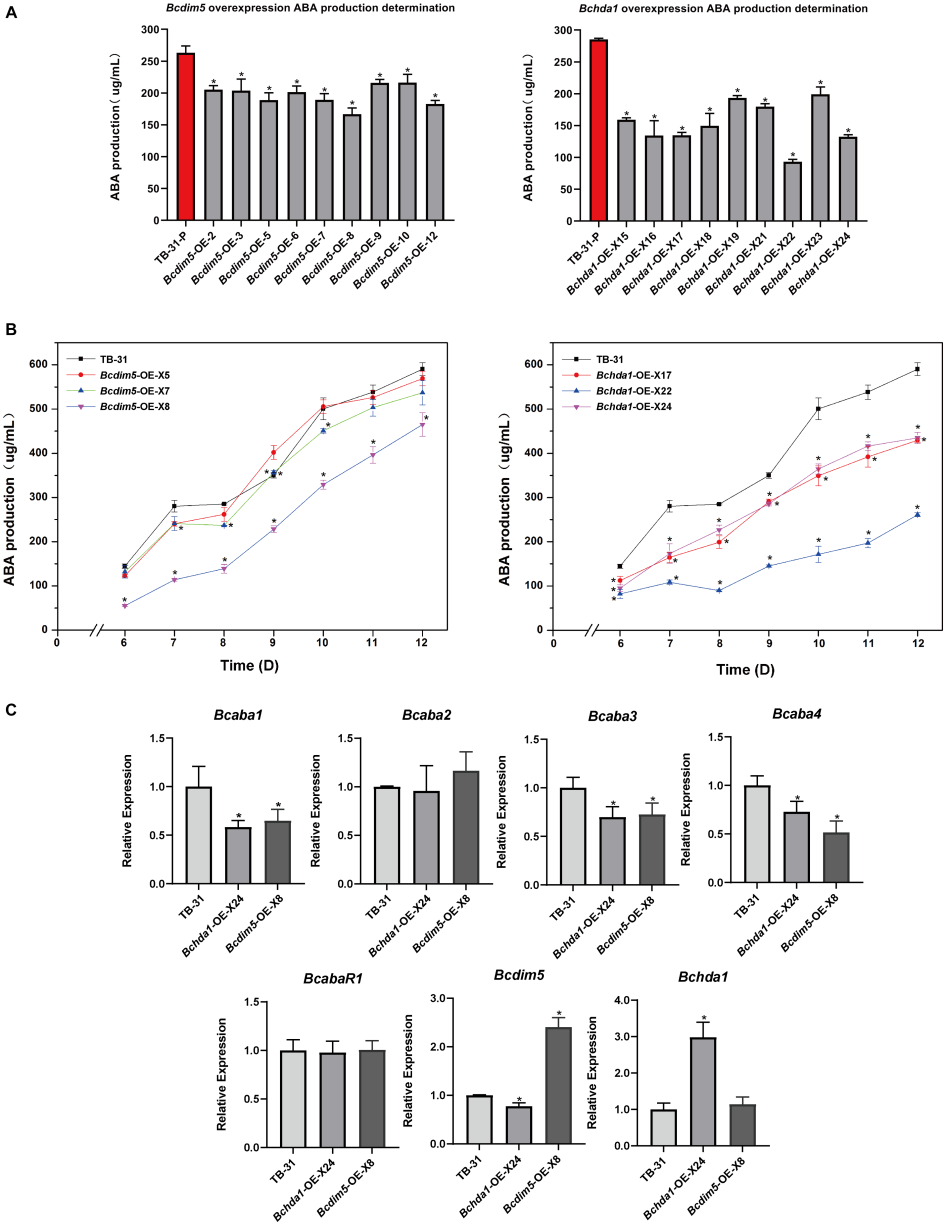
Overexpression of *Bcdim5* and *Bchda1* did not increase ABA production in *B. cinerea* TB-31. **(A)** ABA yield of *Bcdim5* and *Bchda1* overexpression strains cultured for 7 days. The error bars indicate the standard errors of the mean for three replicate cultures (*n* = 3). Asterisks indicate significant differences in ABA production between selected mutants and TB-31 (*p* < 0.05). **(B)** ABA production of control strain TB-31, three randomly selected *Bcdim5*-OE mutants and three randomly selected *Bchda1*-OE mutants at different culture time. Samples for the quantitative determination of ABA production were collected at 6–12 days. The error bars indicate the standard errors of the mean for three replicate cultures (*n* = 3). Asterisks indicate significant differences in ABA production between selected mutants and TB-31 (*p* < 0.05). **(C)** RT-qPCR examining the transcriptional levels of ABA gene cluster, *BcabaR1*, *Bcdim5* and *Bchda1* in *Bcdim5*-OE-X8, *Bchda1*-OE-X24, and TB-31. The relative values for selected genes transcription at 6 days in TB-31 were assigned as 100%. Shown are means and SEM, *n* = 3 independent biological replicates. *p < 0.05 versus the same genes of the TB-31 group.

To further investigate whether the overexpression of *Bchda1* and *Bcdim5* can enhance ABA production, we generated overexpressed mutants and assessed ABA production in 9 randomly selected mutants after 7 days of incubation. The results showed that the ABA production of overexpressed strains was generally reduced (Figure 3A). In order to investigate the potential for ABA production to match or exceed that of the control strain TB-31 during later stages of culture, three *Bchda1* and *Bcdim5* overexpression mutants were randomly selected for 6–12 days to determine the ABA production (Supplementary Figure S4), but the ABA yield was still reduced (Figure 3B).

RNA-seq analysis of ABA synthesis related pathways revealed that a large number of genes related to carbon metabolism was upregulated in *Bchda1*-OE-X24, which was beneficial for ABA synthesis. The increased transcription level of acetoacetyl-CoA synthetase (BCIN_07g02750) in the MVA pathway, enhancing the ability of acetyl-CoA to synthesize acetoacetyl-CoA. Otherwise, the expression changes of related genes in the remaining pathways were not significant in the *Bchda1*-OE-X24 (Supplementary Table S2). Additionally, no significant changes in expression were observed in any of the genes involved in ABA synthesis-related pathways in *Bcdim5*-OE-X8 strain. However, a significant decrease in the FPKM of the ABA cluster was noted in *Bchda1*-OE-X24 and *Bcdim5*-OE-X8 mutants, and further analysis via RT-qPCR revealed reduced expression levels of *Bcaba1*, *Bcaba3* and *Bcaba4* within the cluster (Figure 3C). Therefore, we suggested that the appropriate expression levels of *Bcdim5* and *Bchda1* are important factors for maintaining stable ABA synthesis.

### BcHda1 and BcDIM5 are essential for the normal distribution of H3K9me3 occupancy

**Figure 4:**
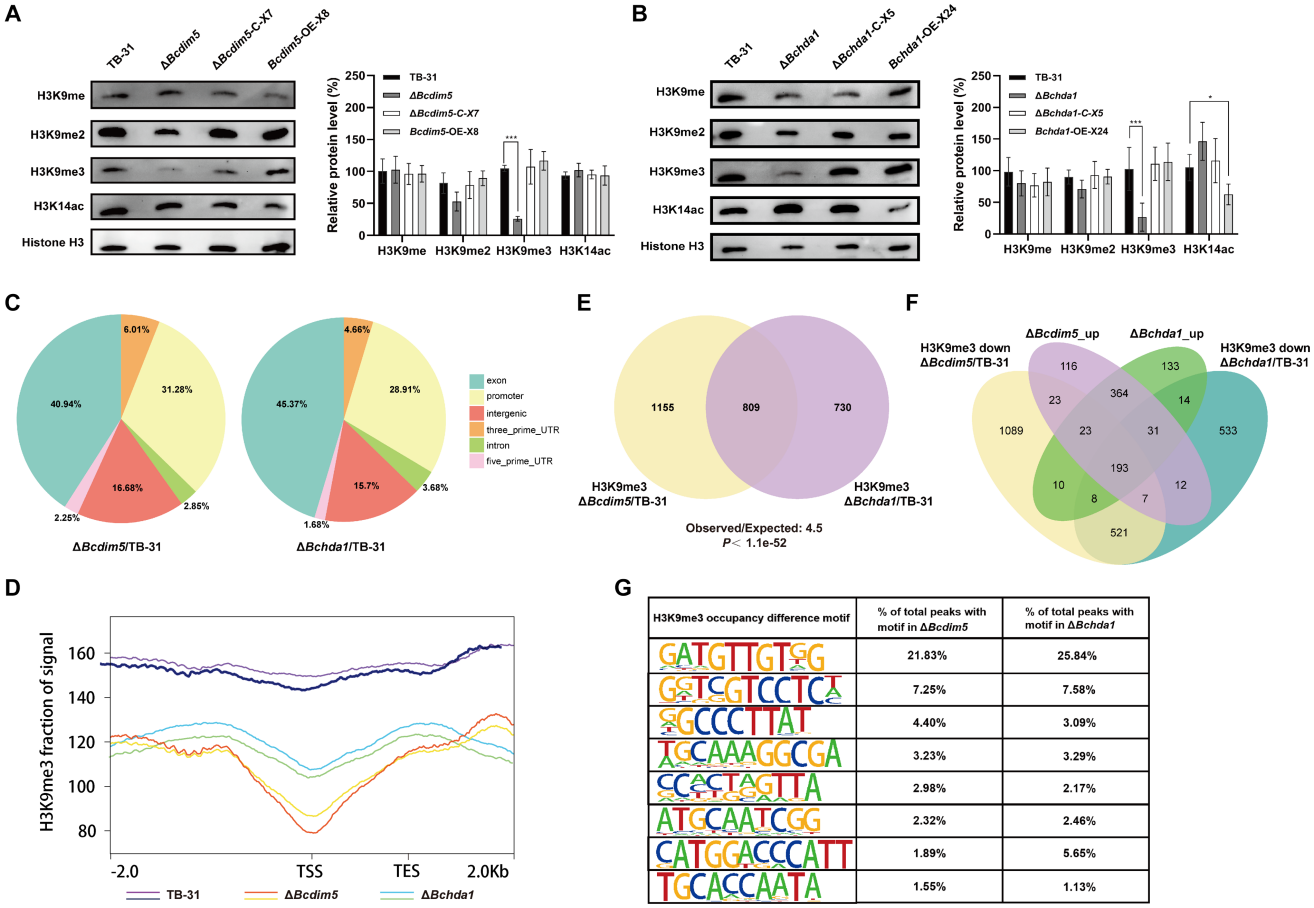
*Bcdim5* and *Bchda1* are essential for the normal distribution of genome-wide H3K9me3 occupancy in *B. cinerea* TB-31. **(A,B)** Global H3K9 methylation statues and H3K14 acetylation statues of the control strain TB-31, *Bcdim5* gene mutants and *Bchda1* gene mutants after dark cultivation for 6 days. Immunoblot signals relative to the TB-31 (set as 1) were quantified. Each value represents the mean ± SE of three biological replicates. The asterisks indicate significant differences between mutants and the TB-31 (*p < 0.05, ***p < 0.001). **(C)** Distribution of BcDIM5 and BcHda1 mediated H3K9me3 removal sites in different regions of annotated genes. The intergenic region refers to the region beyond 2000 bp of the transcription start site of the coding gene and 1,000 bp of the transcription stop site, including the untranscribed and post-transcriptional untranslated regions. **(D)** Metagene plot shows H3K9me3 ChIP-seq intensities within 2.0-kb genomic regions flanking the peak summits at the TSS in the TB-31, Δ*Bcdim5* and Δ*Bchda1* strains. **(E)** Venn diagram showing the significant overlap of H3K9me3-occupied genes in the Δ*Bcdim5* and Δ*Bchda1*. *p*-values for the significance of the overlap between gene sets were calculated using Fisher’s exact test. **(F)** Venn diagram showing the significant overlap between upregulated genes and H3K9me3-occupied genes in the Δ*Bcdim5* and Δ*Bchda1*. **(G)** H3K9me3 occupancy difference motif analysis in the Δ*Bcdim5* and Δ*Bchda1* strains. The 8 most abundant motifs are listed.

Since BcDIM5 is a histone H3K9 methyltransferase and BcHda1 is a putative histone H3K14 deacetylase, their deletion and overexpression will inevitably affect the change of global chromatin modification status. The results showed that H3K9me and H3K9me2 did not change significantly in *Bcdim5* and *Bchda1* mutants. However, the levels of H3K9me3 modification were considerably reduced in Δ*Bchda1* and Δ*Bcdim5*. The restoration of modification levels after gene complementation suggested that BcDIM5 and BcHda1 play a role in regulating the global H3K9me3 modification level. Consequently, it is hypothesized that the alteration of the global H3K9me3 modification state is the primary factor in regulating the DEGs shared by *Bcdim5* and *Bchda1* mutants. Meanwhile, we found that *Bchda1* overexpression resulted in a decrease in the global H3K14ac modification level. The results suggested that BcHda1 functions as a histone H3K14 deacetylase, thereby influencing the global H3K14ac modification level (Figures 4A,B).

The H3K9me3 and H3K14ac play crucial roles in fungal development, virulence, and SMs (Gu et al., 2017; Chen et al., 2022; Zhang et al., 2023), with the former typically associated with gene repression (Gessaman and Selker, 2017) and the latter with gene activation (Wang et al., 2017). In order to gain a deeper understanding of the impact of *Bchda1* and *Bcdim5* loss on H3K9me3 and H3K14ac occupancy in strains, ChIP-seq was conducted to map the genomic distribution of these modifications in the Δ*Bcdim5* and Δ*Bchda1* mutants, with *B. cinerea* TB-31 serving as a background control (Supplementary Dataset S2). The results showed that the level of H3K14ac was almost unchanged in the Δ*Bcdim5* compared to TB-31. In contrast, the Δ*Bchda1* exhibited a higher occupancy of H3K14ac in comparison to TB-31. However, the identification of only 128 H3K14ac differential peaks corresponding to 105 genes indicated that the enriched regions accounted for a minute proportion of the entire genome. This suggested that the loss of *Bchda1* had a relatively minor impact on global H3K14ac. Notably, both the Δ*Bchda1* and Δ*Bcdim5* mutants demonstrated a significant deficiency in the distribution of H3K9me3 occupancy when compared to TB-31. Specifically, the H3K9me3 levels of 998 genes in the Δ*Bchda1* and 1,191 genes in the Δ*Bcdim5* were reduced by at least one-fold. The BcHda1 and BcDIM5 co-mediated H3K9me3 removal sites were distributed in different regions of annotated genes, including exons, promoters, introns, and intergenic regions, with a larger proportion of exons and promoters (Figure 4C). Meta-gene analysis also showed decreased H3K9me3 levels in Δ*Bcdim5* and Δ*Bchda1* strains, especially between TSS and TES regions (Figure 4D). In addition, the additional regions with 254 genes and 80 genes gained H3K9me3 occupancy in the Δ*Bchda1* and Δ*Bcdim5* strains, respectively, but were absent in the TB-31 (Supplementary Dataset S2). Collectively, the loss of BcDIM5 and BcHda1 led to severe global defects in the normal distribution and level of H3K9me3 modification, including majority loss and reduction, and local hypermethylation of genomic sites in *B. cinerea* TB-31.

### Loss of BcHda1 and BcDIM5 reprograms H3K9me3-occupied gene expression

To further compare H3K9me3 distribution between the Δ*Bcdim5* and Δ*Bchda1* strains, we quantified the detailed numbers of H3K9me3-occupied genes with corresponding peaks. In total, we identified 2,846 H3K9me3-occupied peaks (Δ*Bcdim5* to TB-31) corresponding with 1964 genes in the Δ*Bcdim5* and 2069 peaks (Δ*Bchda1* to TB-31) corresponding with 1,539 genes in the Δ*Bchda1*. Among them, 809 genes had overlapping H3K9me3-occupied peaks (Figure 4E), implying that the modification of H3K9me3-enriched regions of these genes may be co-regulated by BcHda1 and BcDIM5. Subsequently, we explored the biological connection between BcHda1 and BcDIM5 in gene repression. RNA-seq analysis indicated that DEGs of Δ*Bcdim5* and Δ*Bchda1* strains showed the same direction of dysregulation with significant overlap in upregulated genes (611 upregulated genes). Meanwhile, 193 genes in the upregulated cross set have H3K9me3 modifications missing or reduced in Δ*Bcdim5* and Δ*Bchda1* strains, indicating that BcDIM5 and BcHda1 are required for transcriptional silencing of these genes (Figure 4F). In addition, 158 genes in the upregulated cross set were adjacent to the H3K9me3 deletion peak domains, suggesting that the spread of H3K9me3 may be required for gene repression. These results indicated that the BcDIM5 and BcHda1 are critical for H3K9me3-mediated transcriptional silencing in *B. cinerea* TB-31. To gain more insights into the characterization of genome-wide H3K9me3 modification of *B. cinerea* TB-31 by BcDIM5 and BcHda1, the motifs of H3K9me3-occupied peaks were predicted and analyzed using HOMER software. The results showed that the predominant motif in abundance is GTTGTNG, followed by GTCCTC, GCCCTT and GGCGA, etc. (Figure 4G). The binding of H3K9me3 on these motifs may require the co-participation of BcDIM5 and BcHda1.

**Figure 5:**
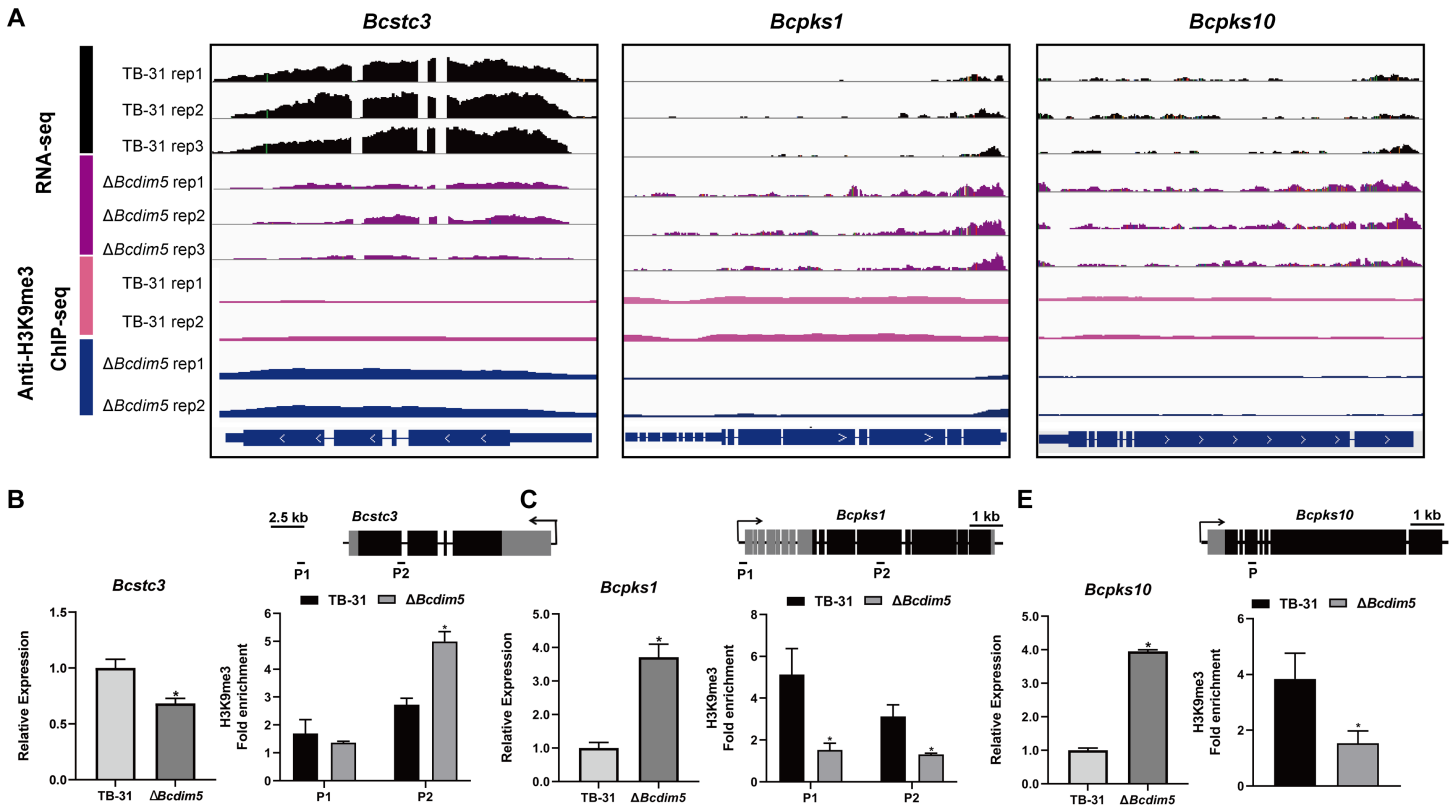
Differential H3K9me3 occupancy due to *Bcdim5* deletion leads to changes in the expression of SM-related genes. **(A)** Genome tracks of RNA-seq, ChIP-seq with anti-H3K9me3 for *Bcstc3*, *Bcpks1* and *Bcpks10* loci in the TB-31 and Δ*Bcdim5* strains. Structures of *Bcstc3*, *Bcpks1* and *Bcpks10* are shown. **(B)** *Bcstc3*; **(C)** *Bcpks1*; **(D)** *Bcpks10*. [i in **(B–D)**] qRT-PCR analysis of *Bcstc3*, *Bcpks1* and *Bcpks10* expression levels. The *B. cinerea* tubulin gene (BC1G_05600) was used as an internal control. The relative values for selected genes transcription at 6 days in TB-31 were assigned as 100%. Shown are means and SEM, *n* = 3 independent biological replicates. *p < 0.05 versus the same genes of the TB-31 group. [ii in **(B–D)**] ChIP-qPCR analysis of H3K9me3 status at the *Bcstc3*, *Bcpks1* and *Bcpks10* loci. IgG was used as a negative control for comparison with IP to calculate the enrichment ploidy. Schematic genetic structures of *Bcstc3*, *Bcpks1* and *Bcpks10*, represented as black boxes for exons, gray boxes for 5′ and 3′ untranslated regions and black lines for promoters and introns. Amplified regions are indicated below each locus (P). Data are presented as the mean ± SD (*n* = 3). *p < 0.05 versus the H3K9me3 occupancy of the TB-31 group.

Histone modification changes induced by BcDIM5 and BcHda1 also affect the transcription levels of SM key enzyme genes of *B. cinerea* (Supplementary Table S2). The transcription levels of *Bcstc1*, *Bcstc3*, *Bcpks11*, *Bcpks17*, and *Bcpks21* genes were decreased in Δ*Bcdim5*, while the transcription levels of *Bcstc4*, *Bcpks1*, *Bcpks10*, and *Bcpks14* genes were increased. In conjunction with ChIP-seq analysis, it was observed that the Δ*Bcdim5* exhibited an increase in H3K9me3 occupancy in the exon region of the *Bcstc3*, while a decrease in H3K9me3 occupancy in the exon region of the *Bcpks1* and *Bcpks10* was noted (Figures 5A–D). Additionally, the transcription levels of five polyketide synthases *Bcpks6, Bcpks9*, *Bcpks11*, *Bcpks1*, and *Bcpks21* genes were all increased in *Bchda1*-OE-X24 strain. The ChIP-qPCR analysis revealed an increase in H3K14ac occupancy in the promoter region of the *Bcpks6* and *Bcpks21* genes (Supplementary Figure S5), which may account for the observed up-regulation of transcription. Histone modification reprogramming caused by BcDIM5 and BcHda1 facilitated the subsequent mining of novel secondary metabolites in *B. cinerea*.

### Deletion of BcHda1 and BcDIM5 causes hyper-H3K9me3 methylation of the ABA gene cluster

**Figure 6:**
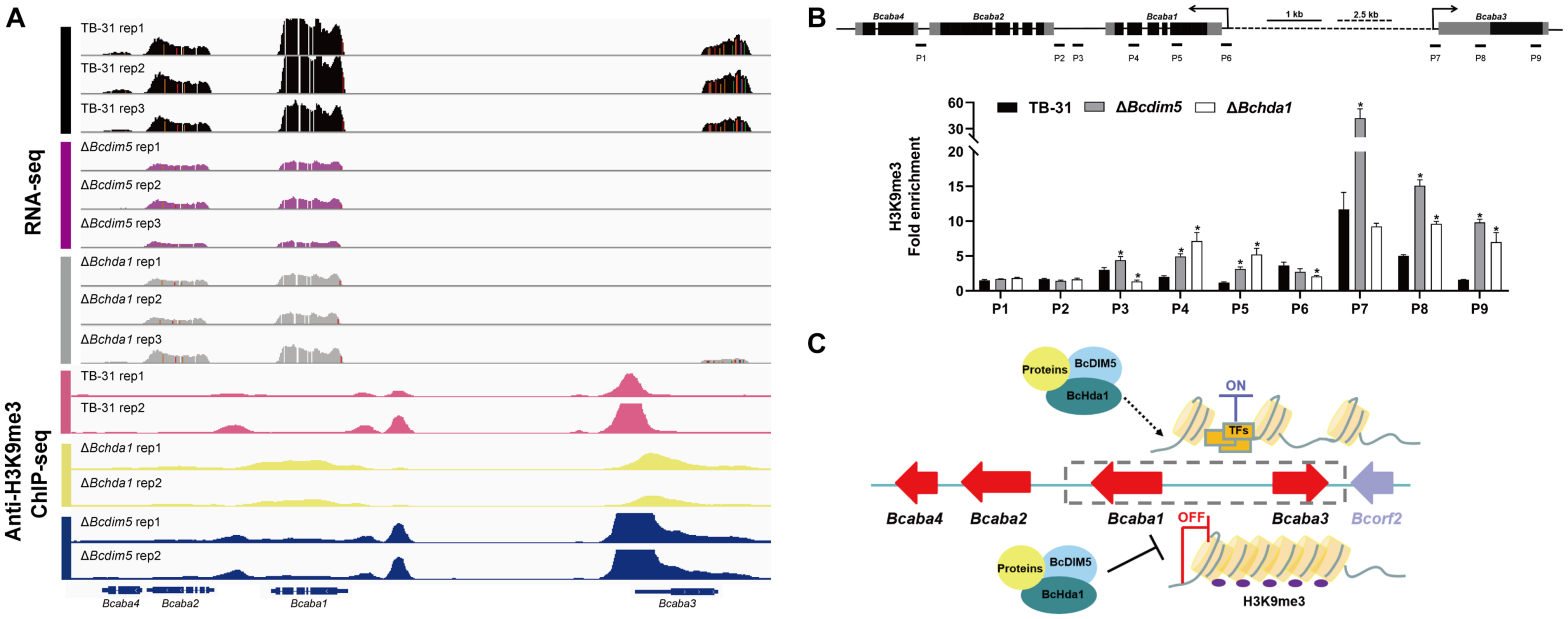
Reduced expression levels of the ABA gene cluster are associated with increased H3K9me3 occupancy. **(A)** Genome tracks of RNA-seq, chromatin immunoprecipitation followed by sequencing (ChIP-seq) with anti-H3K9me3 for ABA gene cluster (*Bcaba1-4*) loci in the TB-31, Δ*Bcdim5* and Δ*Bchda1* strains. Structures of *Bcaba1-4* are shown. **(B)** ChIP-qPCR analysis of H3K9me3 methylation status at the ABA gene cluster loci. IgG was used as a negative control for comparison with IP to calculate the enrichment ploidy. Schematic genetic structures of *Bcaba1*, *Bcaba2*, *Bcaba3* and *Bcaba4*, represented as black boxes for exons, gray boxes for 5′ and 3′ untranslated regions and black lines for promoters and introns. Amplified regions are indicated below each locus (P1-P9). Data are presented as the mean ± SD (*n* = 3). *p < 0.05 versus the H3K9me3 occupancy of the TB-31 group. **(C)** A proposed model to explain the role of BcDIM5 and BcHda1 in regulating ABA biosynthesis in *B. cinerea* TB-31.

Current studies suggest that H3K9me3 is commonly associated with the repression of biosynthetic gene clusters, and has been further identified as a mark of constitutive heterochromatin, principally associated with centromeric heterochromatin (Wiles and Selker, 2017; Torres-Garcia et al., 2020). Therefore, knockdown of DIM5 in pathogenic fungi leads to increased production of some secondary metabolites (Wang et al., 2022). However, DIM5 is often associated with the pathogenicity of the strain. The loss of DIM5 is accompanied by slow growth and development of strains, weakened toxicity, and reduced virulence factor synthesis (Gu et al., 2017), which also occurs in *B. cinerea* (Zhang et al., 2016). In the Δ*Bcdim5* and Δ*Bchda1* mutants, the production of the virulence factor ABA was severely impaired. IGV analysis of the H3K14ac and H3K9me3 enrichment of ABA gene clusters in TB-31, Δ*Bcdim5*, and Δ*Bchda1* revealed that the H3K14ac modification did not exhibit a significant difference. Unexpectedly, the deletion of *Bcdim5* and *Bchda1* did not result in a decrease in H3K9me3 occupancy within the ABA gene cluster, but rather a hyper-H3K9me3 enrichment (Figure 6A). Subsequently, ChIP-qPCR was used to further validate that H3K9me3 occupancy in the promoter region and exon region of *Bcaba1* and *Bcaba3* genes increased significantly (Figure 6B), which in turn led to the down-regulation of the expression of the entire gene cluster. Whether the generation of this hyper-H3K9me3 modification is due to the deletion of *Bcdim5* and *Bchda1* resulting in the recruitment of other histone-modifying enzymes to the ABA gene cluster needs to be further investigated. However, there is no doubt that BcDIM5 and BcHda1 are required for ABA synthesis. Meanwhile, we also found that BcDIM5 and BcHda1 did not directly bind to the promoter region of the ABA gene cluster by electrophoretic mobility shift assays (data not shown). Therefore, we believe that other proteins assist BcDIM5 and BcHda1 in the H3K9me3 modification of ABA gene clusters (Figure 6C).

**Figure 7:**
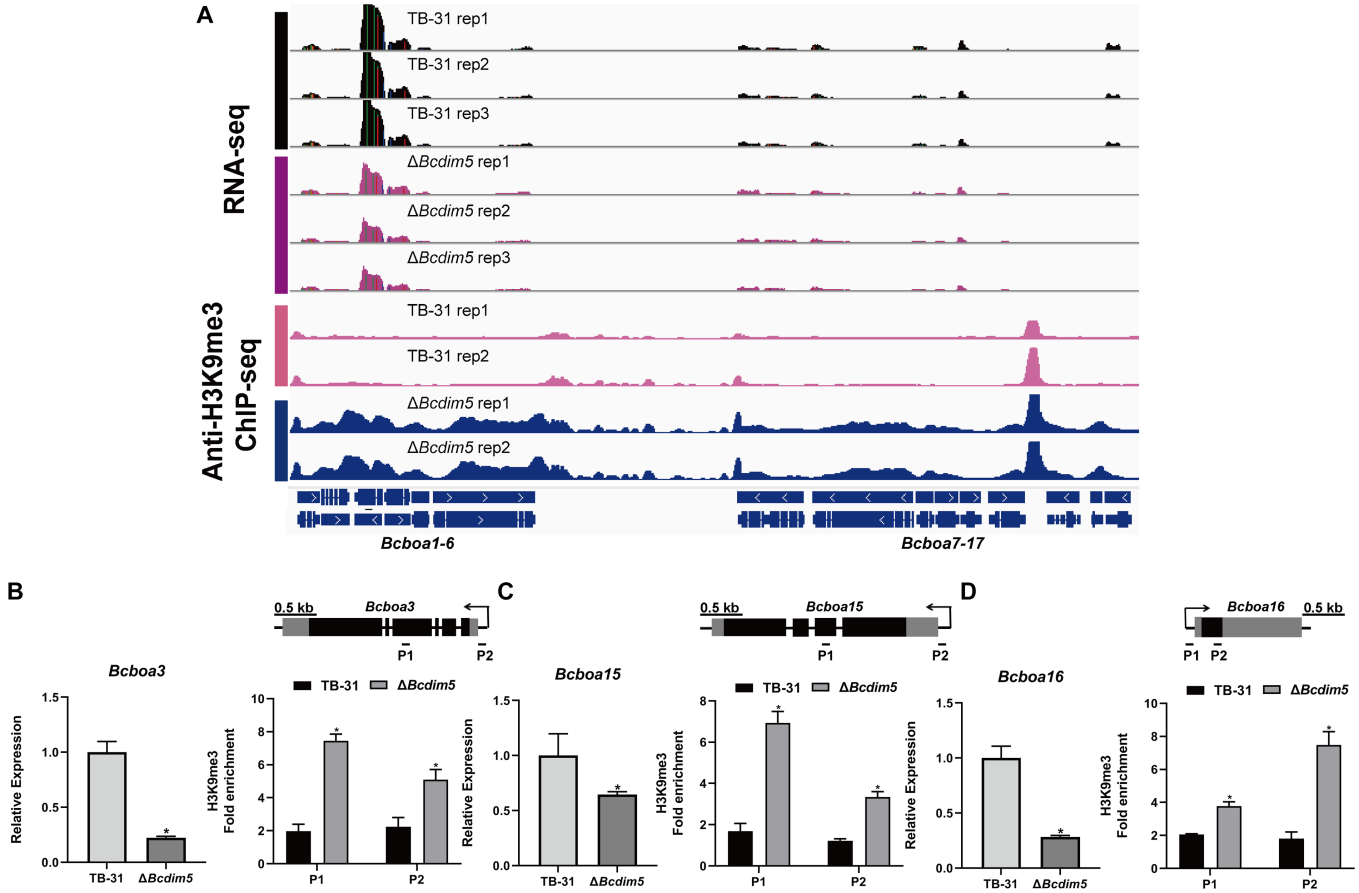
Reduced expression levels of the BOA gene cluster are associated with increased H3K9me3 occupancy. **(A)** Genome tracks of RNA-seq, ChIP-seq with anti-H3K9me3 for BOA gene cluster (*Bcboa1-17*) loci in the TB-31 and Δ*Bcdim5* strains. Structures of *Bcboa1-17* are shown. **(B)** *Bcboa3*; **(C)** *Bcboa15*; **(D)** *Bcboa16*. [i in **(B–D)**] qRT-PCR analysis of *Bcboa3*, *Bcboa15*, *Bcboa16* expression levels. The *B. cinerea* tubulin gene (BC1G_05600) was used as an internal control. The relative values for selected genes transcription at 6 days in TB-31 were assigned as 100%. Shown are means and SEM, *n* = 3 independent biological replicates. *p < 0.05 versus the same genes of the TB-31 group. [ii in **(B–D)**] ChIP-qPCR analysis of H3K9me3 status at the *Bcboa3*, *Bcboa15*, *Bcboa16* loci. IgG was used as a negative control for comparison with IP to calculate the enrichment ploidy. Schematic genetic structures of *Bcboa3*, *Bcboa15*, *Bcboa16*, represented as black boxes for exons, gray boxes for 5′ and 3′ untranslated regions and black lines for promoters and introns. Amplified regions are indicated below each locus (P1, P2). Data are presented as the mean ± SD (*n* = 3). *p < 0.05 versus the H3K9me3 occupancy of the TB-31 group.

Apart from the ABA gene cluster, we conducted an analysis of two other well-known virulence factors (Simon et al., 2013; Porquier et al., 2019), namely BOT (botrydial) and BOA (botcinic acid). RNA-seq data revealed that the expression levels of the BOA and BOT gene clusters remained unaltered in the Δ*Bchda1* strain. However, in the Δ*Bcdim5* strain, the expression levels of *Bcboa3*, *Bcboa15*, *Bcboa16*, *Bcboa17* and the entire BOT gene cluster (*Bcbot1-5*) were downregulated. IGV showed increased H3K9me3 occupancy in the exon and promoter regions of the *Bcboa3*, *Bcboa15*, and *Bcboa16* genes in Δ*Bcdim5* strain, which may be responsible for the down-regulation of expression (Figures 7A–D). Notably, the BOT gene cluster did not exhibit any significant change in H3K9me3 occupancy (Supplementary Figure S6), so we suggested that the knockdown of *Bcdim5* indirectly led to the down-regulation of transcription levels in the whole cluster. Deletion of *Bcdim5* and *Bchda1* disrupted the normal post-translational modifications of some virulence factors (ABA, BOA), which also reflected the complexity of the regulatory network of virulence factors. Combined with the analysis of the above studies, we concluded that the ABA gene cluster is co-regulated by BcDIM5 and BcHda1, and they are essential for the normal distribution and maintenance of the *B. cinerea* TB-31 ABA gene cluster H3K9me3.

## Discussion

The effect of histone modification on the development and virulence of the common plant pathogenic fungus *B. cinerea* has been widely reported. However, the effect of this epigenetic modification on the synthesis of the virulence factor ABA in *B. cinerea* remains unclear. Here, we conducted an investigation on the H3K9 methyltransferase BcDIM5. Our findings indicate that knockdown of *Bcdim5* led to a decrease in the overall expression level of the ABA gene cluster in *B. cinerea* TB-31, resulting in impaired ABA synthesis. Additionally, we identified a histone deacetylase BcHda1 that physically associates with BcDIM5, and both proteins play crucial roles in maintaining transcriptional silencing and normal distribution of H3K9me3 in *B. cinerea* TB-31. This work further extends our understanding of the complex regulatory network of ABA biosynthesis.

Deletion of the histone methyltransferase BcDIM5 has been reported to result in silencing of the pathogenic genes and to reduce the virulence of *B. cinerea* B05.10 (Zhang et al., 2016), which is consistent with the conclusions we reached. When BcDIM5 was knocked down, global H3K9me3 modification levels were reduced. As important virulence factors of *B. cinerea*, the reduced production of ABA and BOA in Δ*Bcdim5* will inevitably affect the virulence of the strain. In another study, Reyez-Dominguez and colleagues demonstrated that the loss of H3K9me3 caused a decrease in DON synthesis in *F. graminearum*, which hindered the transmission of the fungus from infected flowering wheat heads to neighboring spikelets (Etier et al., 2022). In the mango pathogen *Fusarium mangiferae*, the loss of H3K9 methyltransferase *kmt1* almost completely inhibits the biosynthesis of the toxins fusapyrone and deoxyfusapyrone (Atanasoff-Kardjalieff et al., 2021). All these results indicate that H3K9 methyltransferase is closely related to virulence factors of strains. Therefore, we suggest that when H3K9 methyltransferase is deleted, rapid changes in the H3K9me3 modification pattern lead to a coordinated interaction between fungal development and virulence, affecting the normal post-translational modification (PTM) of some virulence factors.

Normal genome-wide H3K9me distribution is essential for both pathogens and symbionts in fungi–host interactions (Lai et al., 2022). In the present study we found that the genome-wide H3K9me3 occupancy distribution after *Bchda1* and *Bcdim5* loss showed significant defects, with 998 genes in the Δ*Bchda1* and 1,191 genes in the Δ*Bcdim5* showing at least a 1-fold decrease in H3K9me3 levels. Meanwhile RNA-seq and ChIP-seq data has revealed a significant intersection of DEGs and H3K9me3 differential peaks when *Bchda1* and *Bcdim5* were lost compared to TB-31. At least 193 genes were up-regulated in Δ*Bcdim5* and Δ*Bchda1* strains due to the loss or reduction of H3K9me3 modification, implying that BcHda1 and BcDIM5 would jointly maintain a normal distribution of H3K9me3 modifications.

Due to the versatile of the histone code, two different types of histone modification crosstalk have been proposed in yeast. The cis-effect is described as a “communication” of adjacent modifications within the same histone tails (Fischle et al., 2003). For example, in fission yeast, Clr4 has been shown to associate with the ubiquitin E3 ligase Cul4, forming the Clr4 methyltransferase complex (Oya et al., 2019). This complex preferentially ubiquitinates H3K14 and then promotes the methylation of H3K9. Trans-effects design cross-communications between different histone marks located within the same nucleosome (Fingerman et al., 2008). For example, H2BK123ub is essential for the deposition of H3K4me2/me3 and H3K79me2/me3, which in turn affects gene expression (van Welsem et al., 2018). In contrast, in the present study, H3K14ac did not change after *Bcdim5* loss, indicating that *Bcdim5* is not involved in the global regulation of H3K14ac modification. The enriched regions generated by H3K14ac modification after *Bchda1* loss accounted for a very small proportion of the entire genome, with only 105 genes having altered H3K14ac occupancy. However, we found that global H3K14ac modification decreased when *Bchda1* was overexpressed, suggesting that the function of its own deacetylase is only exercised when the normal distribution of H3K9me3 is steady. This may be a unique regulatory mechanism and is deserving of further research. In addition to ABA, BcDIM5 and BcHda1 also cause changes in transcription levels of other key enzymes of SM, most of which correspond to compounds not reported in the literature, which provides favorable support for the subsequent excavation of unknown SMs in *B. cinerea*.

In conclusion, our results identified a physical linkage between the H3K9 methyltransferase BcDIM5 and the H3K14 deacetylase BcHda1, revealing their regulatory roles in ABA biosynthesis. BcDIM5 and BcHda1 play a key role in the normal distribution of H3K9me3 occupancy and the stable synthesis of SMs in *B. cinerea* TB-31. To our knowledge, this is the first report on the involvement of histone modifications in *B. cinerea* ABA biosynthesis. This work provides a theoretical basis for further genetic improvement of high-yielding ABA strains and new inspiration for the discovery and production of unknown SMs in *B. cinerea*.

## Materials and methods

### Strains, plasmids, and molecular biology experiments

In our laboratory, the *B. cinerea* wild-type strain TBC-6 was isolated from wheat stems and leaves in southwest China (Tan et al., 1998). The ABA-hyper producing *B. cinerea* mutant TB-31 was generated from multiple rounds of mutagenesis and screening initiated from *B. cinerea* TBC-6 (Gong et al., 2014). Binary vectors pCBh1, pCBg1, and pCBsilent1 used in this study were constructed by our laboratory (Ding et al., 2015). After conidia maturation, *Agrobacterium tumefaciens* EHA105-mediated transformation (ATMT) was performed as previously described (Rolland et al., 2003). The detailed operation of ATMT, extracellular ABA quantification, quantitative RT-PCR, histone extraction analysis and DNA/RNA extraction were performed as previously described (Wei et al., 2022).

### Construction of transformants

A list of all primers used to prepare transformants and RT-qPCR is shown in Supplementary Table S1.

Briefly, the knockout transformants were constructed using a double-combined PCR method (Yu et al., 2004). The upstream fragment and downstream fragment of *Bcdim5* ORF were amplified with primer pairs *Bcdim5*-5-F1/−R1 and *Bcdim5*-3-F1/−R1, respectively. The hygromycin expression cassette fragment (*PoliC::hph*) was then amplified using the primer pair Hph-F1/Hph-R1. The knockout cassette was obtained by overlapping PCR with primer pair *Bcdim5*-5-F1/*Bcdim5*-3-R1, and then transformed into TB-31 protoplasts. The *Bchda1* knockout mutant was constructed in the same way as above. The protoplasts were produced as previously described (Choquer et al., 2008). The construction of complement transformants and overexpression transformants (promoter: *Aspergillus nidulans oliC*) was carried out by ATMT transformation method to transfer target vectors into corresponding strains. Transformants were selected on PDA containing hygromycin (50 μg/mL) or glyphosate (100 μg/mL) by three rounds of subcultures with the same antibiotic selection.

### RNA-seq

Undifferentiated hyphae of *B. cinerea* TB-31, Δ*Bchda1*, Δ*Bcdim5*, *Bchda1*-OE-X24, *Bcdim5*-OE-X8 transformants were identified, and the hyphae were harvested for RNA extraction after dark cultivation on potato dextrose agar (PDA) plates at 25°C for 6 days. RNA quality was assessed on an Agilent 2,100 Bioanalyzer (Agilent Technologies, Palo Alto, CA, USA) and checked using RNase free agarose gel electrophoresis. After total RNA was extracted, eukaryotic mRNA was enriched by Oligo (dT) beads. After total RNA was extracted, prokaryotic mRNA was enriched by removing rRNA by Ribo-ZeroTM Magnetic Kit (Epicentre, Madison, WI, USA). Then the enriched mRNA was fragmented into short fragments using fragmentation buffer and reversly transcribed into cDNA by using NEBNext Ultra RNA Library Prep Kit for Illumina (NEB #7530, New England Biolabs, Ipswich, MA, USA). The purified double-stranded cDNA fragments were end repaired, A base added, and ligated to Illumina sequencing adapters. The ligation reaction was purified with the AMPure XP Beads (1.0X). Ligated fragments were subjected to size selection by agarose gel electrophoresis and poly merase chain reaction (PCR) amplified. The resulting cDNA library was sequenced using Illumina Novaseq 6000 by Gene Denovo Biotechnology Co. (Guangzhou, China).

### Chromatin immunoprecipitation

Chromatin immunoprecipitation (ChIP) was performed as previously described with some necessary modification (Liu et al., 2019). *B. cinerea* strains were grown on potato dextrose agar (PDA) slants at 25°C for 6 days. Mycelia were collected in filtered and clarified 1xPDB liquid medium with 1% (v/v) formaldehyde. Crosslinking was performed at 25°C and 90 rpm for 15 min, then 125 mM glycine was added to terminate fixation, and incubated at room temperature for 5 min after mixing. Subsequently, the treated samples were rinsed with water, frozen and ground in liquid nitrogen for chromatin isolation. Anti-H3K9me3 (Active Motif) and anti-H3K14ac (Active Motif) were used for immunoprecipitation into the chromatin. Immunoprecipitated DNA was used for ChIP followed by sequencing (ChIP-seq) DNA library preparation or ChIP-qPCR analysis.

### ChIP-seq analysis

ChIP analysis was carried out following the previously provided protocol by Wuhan IGENEBOOK Biotechnology Co., Ltd.^1^ (Gendrel et al., 2005). ChIP-seq libraries were sequenced on the Illumina NovaSeq 6000 platform. Raw Data, processed using the software FastQC (version: 0.11.5) for quality control (de Sena Brandine and Smith, 2019). For the annotation of genomic distribution of peaks, we divided the genome into five fragments: promoter [the 2-kb region upstream of the transcription start site (TSS)], exon, intron, transcription termination site (TTS) [the 1-kb region downstream of the transcription end site (TES)] and Intergenic (outside of these regions).

### ChIP-qPCR analysis

ChIP-qPCR was analyzed by Fold Enrichment method. The CT value of IgG was used as a negative control for comparison with IP to calculate the fold enrichment (Zhuang et al., 2022). The abundance of immunoprecipitated chromatin was determined by qRT-PCR using the primers given in Supplementary Table S1. The data are presented as mean ± SD of three biological replicates.

### GST-pulldown

The GST and GST-tag fusion protein were purified by pGEX-6P-1 vector. Use pET28a (+) vector to purify His-tag fusion protein. The constructed vectors were then transferred into *E. coli* Rosetta for expression. The expression of GST and GST-BcDIM5 proteins was induced at 16°C for 24 h (0.5 mM IPTG). The expression of BcHda1 protein was induced at 22°C for 16 h (0.5 mM IPTG). 500 μg purified GST and GST fusion protein were incubated with GST-Tag resin at 4°C for 4 h. Add the interaction His-tag fusion protein 500 μg and rotate overnight at 4°C. The protein was washed with elution buffer at least 7 times, and then use a small amount of eluting buffer to elute the protein for subsequent Western Blot.

### Yeast-two-hybrid assay

To confirm interactions, yeast-two-hybrid analyses were applied using the Matchmaker^®^ Gold Yeast-Two-Hybrid System (Takara Bio USA, Inc.) according to manufacturer’s instructions. *Bcdim5* was inserted into the pGBKT7 bait vector, *Bchda1* was inserted into the pGADT7 prey vector. Bait and prey constructs were transformed into Y2H Gold and Y187 strains as described in instruction for the Matchmaker system, respectively.

### Statistical analysis

Data were expressed as the mean ± SD. Differences among different treatments were compared using SPSS v.16.0 (SPSS Inc., Chicago, IL, United States). *p*-values were calculated using Fisher’s exact test for overlapping using online tools to determine the significance of the overlap of two gene sets.

## Data availability statement

The datasets presented in this study can be found in online repositories. The names of the repository/repositories and accession number(s) can be found in the article/Supplementary material. The data presented in the study are deposited in the “Science Data Bank” repository, accession number: 10.57760/sciencedb.09674.

## Author contributions

ZW: Conceptualization, Investigation, Methodology, Writing – original draft, Writing – review & editing. DS: Funding acquisition, Writing – review & editing. XH: Data curation, Software, Writing – review & editing. TL: Data curation, Software, Writing – review & editing. ZL: Investigation, Project administration, Writing – review & editing. DL: Project administration, Writing – review & editing. JY: Validation, Writing – review & editing. HT: Funding acquisition, Resources, Writing – review & editing.

## Footnotes

^1^ http://www.igenebook.com
