# Supplementary material for: Normal distribution of H3K9me3 occupancy co-mediated by histone methyltransferase BcDIM5 and histone deacetylase BcHda1 maintains stable ABA synthesis in Botrytis cinerea TB-31
Source: Front Microbiol. 2024 Mar 4;15:1339576. doi: 10.3389/fmicb.2024.1339576 (PMC10944950; doi:10.3389/fmicb.2024.1339576)

Frontiers in Microbiology **Supporting Information**

The following Supporting Information is available for this article:

Fig. S1 Construction and validation of knockout mutants.

Fig.S1 Construction and validation of knockout mutants. (a) Schematic diagram of the construction of the knockout mutant. Target gene conserved domain was replaced by hygromycin expression cassette (*PoliC::hph*). Primers labeled for amplification of Hph expression cassette and validation of knockout strains. (b) Diagnostic PCR to validate knock-out results. The primer pairs used in lane 1 is Hph-F1/Hph-R1, the primer pairs used in lane 2 is *dim5, hda1*-F7/Hph-YZ-R1, the primer pairs used in lane 3 is Hph-YZ-F1/*dim5, hda1*-R11, the primer pairs used in lane 4 is *dim5, hda1*-F6/*dim5, hda1*-R10.

(a)

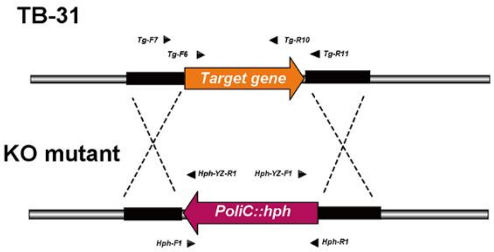

(b)

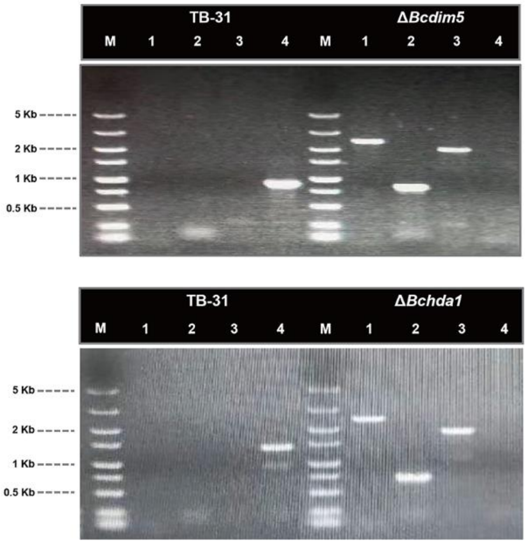

Fig. S2 DEGs analysis of Δ*Bchda1* and Δ*Bcdim5* compared to control strain TB-31.

Fig.S2 DEGs analysis of Δ*Bchda1* and Δ*Bcdim5* compared to control strain TB-31. (a) TB-31 vs Δ*Bcdim5*; (a) TB-31 vs Δ*Bchda1*. Counts of DEGs significantly enriched to GO function classes and KEGG pathways, respectively. The enrichment pathways of the top 20 are shown.

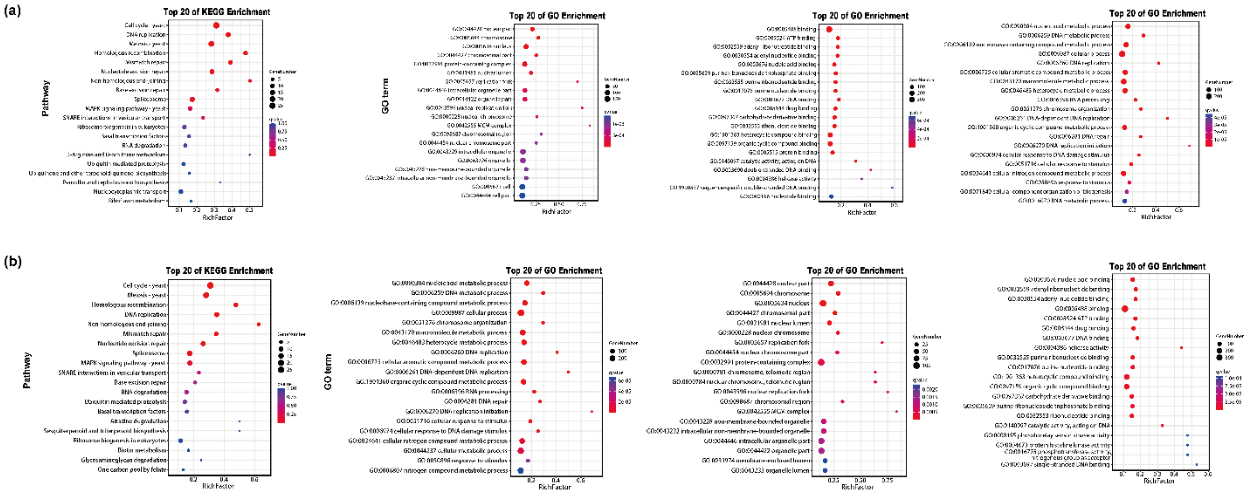

Fig. S3 Deletion of BcDIM5 and BcHda1 inhibits strain growth and development

Fig.S3 Deletion of BcDIM5 and BcHda1 inhibits strain growth and development. The TB-31, BcDIM5 mutants and BcHda1mutants were photographed after 6 days of PDA growth.

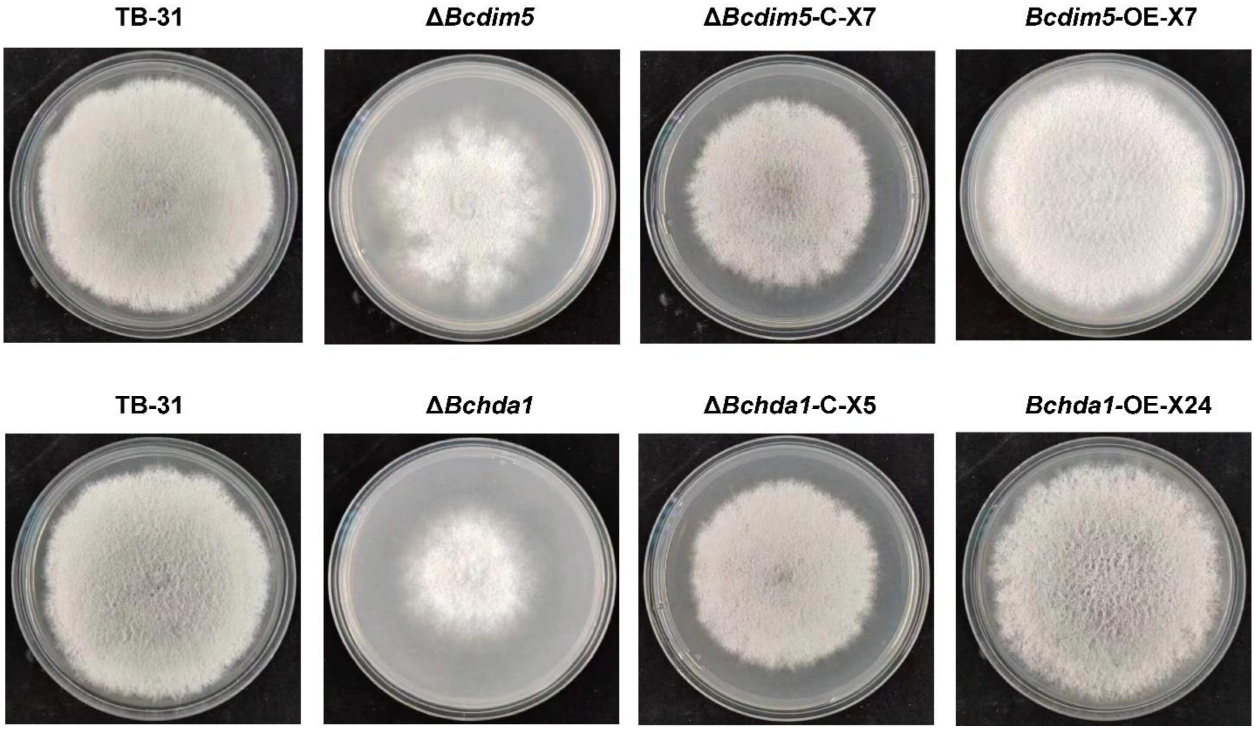

Fig. S4 Validation of *Bcdim5* and *Bchda1* overexpression strains

Fig.S4 Validation of *Bcdim5* and *Bchda1* overexpression strains. RT-qPCR was performed to detect the transcription levels of *Bcdim5* and *Bchda1* in randomly selected *Bcdim5* and *Bchda1* overexpressing strains and TB-31. The relative transcriptional levels of selected genes were obtained after normalization to the constitutive tubulin reference gene (BC1G_05600) at 6 days. The relative values for selected genes transcription at 6 days in TB-31 were assigned as 100%. Shown are means and SEM, n = 3 independent biological replicates. **P* < 0.05 versus the same genes of the TB-31 group.

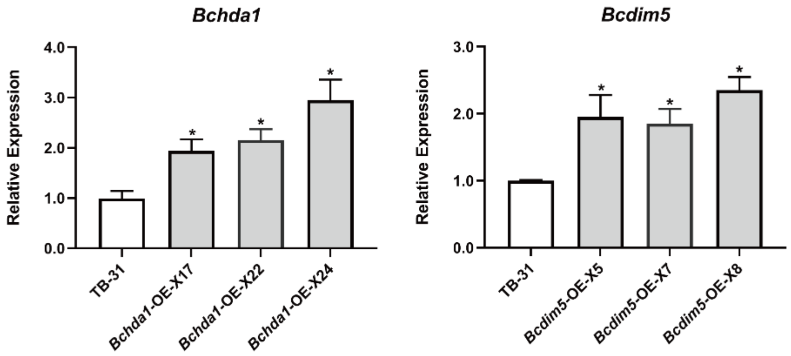

Fig. S5 Overexpression of *Bchda1* leads to changes in H3K14ac occupancy of *Bcpks6* and *Bcpks21.*

Fig.S5 Overexpression of *Bchda1* leads to changes in H3K14ac occupancy and thus affects the expression of *Bcpks6* and *Bcpks21* genes. (a) *Bcpks6*; (b) *Bcpks21.* (i in a, b) qRT-PCR analysis of *Bcpks6* and *Bcpks21* expression levels. The *B.cinerea* tubulin gene (BC1G_05600) was used as an internal control. The relative values for selected genes transcription at 6 days in TB-31 were assigned as 100%. Shown are means and SEM, n = 3 independent biological replicates. **P* < 0.05 versus the same genes of the TB-31 group. (ii in a, b) ChIP-qPCR analysis of H3K14ac status at the *Bcpks6* and *Bcpks21* loci. IgG was used as a negative control for comparison with IP to calculate the enrichment ploidy. Schematic genetic structures of *Bcpks6* and *Bcpks21*, represented as black boxes for exons, gray boxes for 5' and 3' untranslated regions and black lines for promoters and introns. Amplified regions are indicated below each locus (P). Data are presented as the mean ± SD (n = 3). **P* < 0.05 versus the H3K14ac occupancy of the TB-31 group.

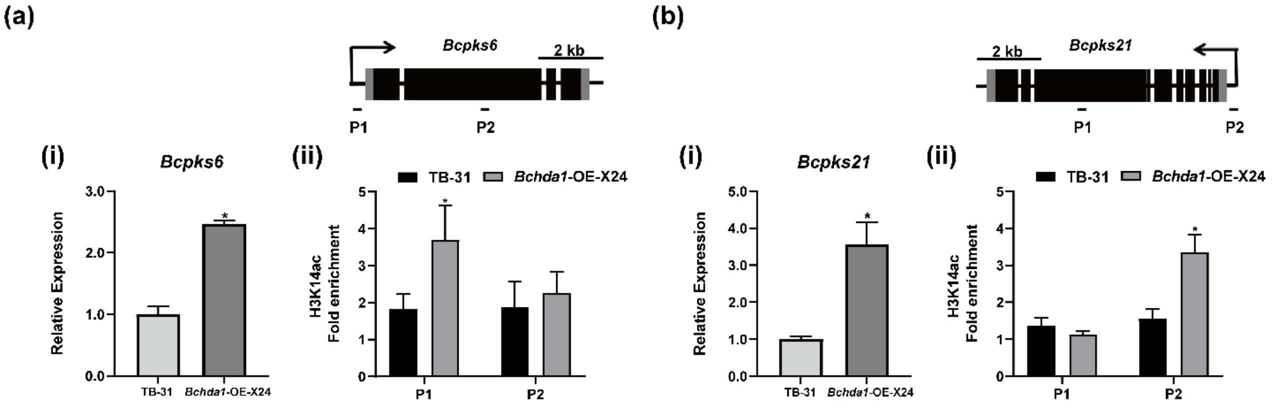

Fig. S6 Down-regulation of BOT gene cluster expression is not directly related to H3K9me3 occupancy.

Fig.S6 Down-regulation of BOT gene cluster expression is not directly related to H3K9me3 occupancy. (a) Genome tracks of RNA-seq, ChIP-seq with anti-H3K9me3 for BOT gene cluster (*Bcbot1-5*) loci in the TB-31 and Δ*Bcdim5* strains. Structures of *Bcbot1-5* are shown. (b) *Bcbot4*; (c) *Bcbot5*. (i in b–d) qRT-PCR analysis of *Bcbo4*, *Bcbot5* expression levels. The *B.cinerea* tubulin gene (BC1G_05600) was used as an internal control. The relative values for selected genes transcription at 6 days in TB-31 were assigned as 100%. Shown are means and SEM, n = 3 independent biological replicates. **P* < 0.05 versus the same genes of the TB-31 group. (ii in b–d) ChIP-qPCR analysis of H3K9me3 status at the *Bcbo4*, *Bcbot5* loci. IgG was used as a negative control for comparison with IP to calculate the enrichment ploidy. Schematic genetic structures of *Bcbo4* and *Bcbot5*, represented as black boxes for exons, gray boxes for 5' and 3' untranslated regions and black lines for promoters and introns. Amplified regions are indicated below each locus (P). Data are presented as the mean ± SD (n = 3). **P* < 0.05 versus the H3K9me3 occupancy of the TB-31 group.

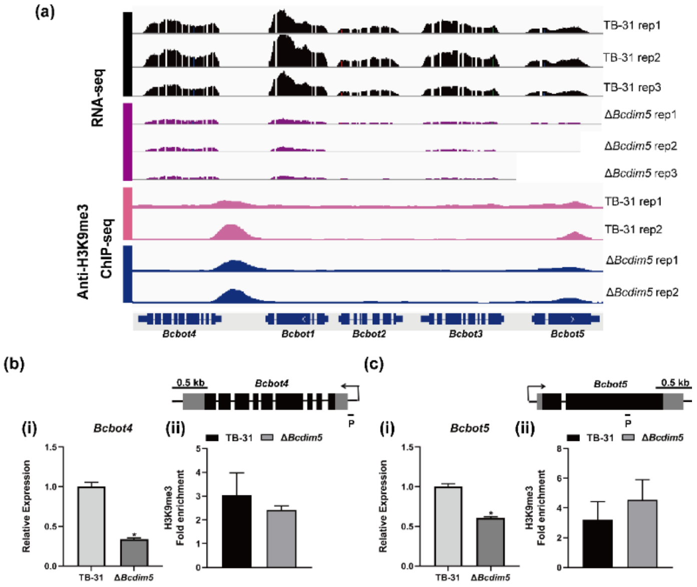

Supplement: Supplementary file 3 [file Data_Sheet_1.pdf]
